# Cachexia vs. Obesity: A Persisting Imbalance Between Clinical Need and Research Effort

**DOI:** 10.1002/jcsm.70372

**Published:** 2026-08-21

**Authors:** Carolyn Blair, Alexander P. Maxwell, Adrian Slee, Clare McKeaveney, Marion Carson, Faizan Awan, Malcolm Brown, Marissa Burgermaster, Andrew Davenport, Damian Fogarty, Denis Fouque, Oonagh Gooding, Kamyar Kalantar‐Zadeh, Karen Magee, Robert Mullan, Helen Noble, Sam Porter, David S. Seres, Joanne Shields, Ian Swaine, Miles Witham, Joanne Reid

**Affiliations:** ^1^ School of Nursing and Midwifery Queen's University Belfast Belfast UK; ^2^ Centre for Public Health Queen's University Belfast Belfast UK; ^3^ Division of Medicine, Faculty of Medical Sciences University College London London UK; ^4^ Renal Unit Antrim Area Hospital, Northern Health & Social Care Trust Antrim UK; ^5^ Chair of Renal Patient Led Advisory Network (RPLAN) Lancashire UK; ^6^ School of Sport and Exercise Science Ulster University Belfast UK; ^7^ Nutritional Sciences and Population Health University of Texas at Austin Austin Texas USA; ^8^ UCL Department of Renal Medicine Royal Free Hospital University College London London UK; ^9^ Regional Nephrology Unit Belfast City Hospital, Belfast Health & Social Care Trust Belfast UK; ^10^ Division of Nephrology, Dialysis and Nutrition Hôpital Lyon Sud and University of Lyon Lyon France; ^11^ Irvine Division of Nephrology, Hypertension and Kidney Transplantation University of California Oakland California USA; ^12^ School of Health and Care Bournemouth University Bournemouth UK; ^13^ Institute of Human Nutrition and Department of Medicine Columbia University Irving Medical Center New York New York USA; ^14^ School of Human Sciences University of Greenwich London UK; ^15^ AGE Research Group, NIHR Newcastle Biomedical Research Centre Newcastle University Newcastle UK

In 2013, von Haehling and Anker [1] published an editorial: ‘Cachexia vs. obesity: where is the real unmet clinical need?’ This editorial highlighted the publication volumes and MeSH descriptor differentials between these clinical entities. ‘Obesity’ was present in titles at a rate of over 25 times that of ‘Cachexia’. Over a decade later, there remains a similar imbalance in the number of publications on obesity as compared to cachexia or wasting disorders. In PubMed, the number of entries that contain ‘cachexia’ as a title word is only 4478, whereas the number of entries with ‘obesity’ in the title is 123 388, giving a ratio of nearly 27 to 1 in favour of ‘obesity’ publications. Although the number of ‘cachexia papers’ has increased substantially from 1825 in 2013, this is dwarfed by the number of entries with ‘obesity’ in the title, and the ratio remains strongly in favour of ‘obesity’ publications. Nonetheless, the discrepancy between the numbers of ‘obesity’ publications and ‘cachexia’ papers is slowly diminishing (Figure 1).

**FIGURE 1:**
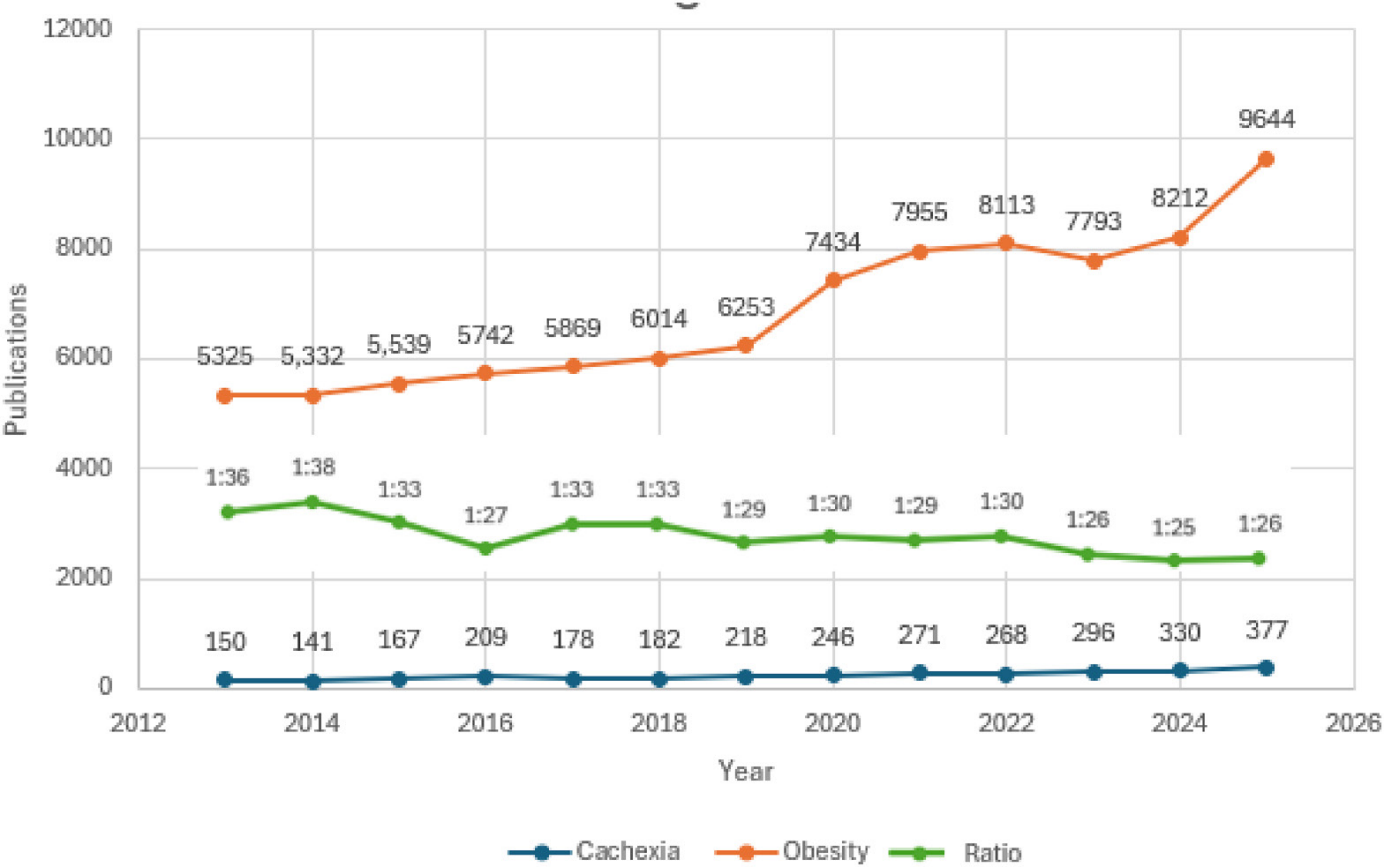
Number of PubMed entries for obesity or cachexia as respective title words with the ratio of cachexia vs. obesity. Assessed on 27 January 2026 from www.pubmed.gov.

There are still very few national or international cachexia guidelines. Indeed, although the search term ‘obesity [title word] AND guideline* [title word]’ yields 220 entries, the opposing search ‘cachexia [title word] AND guideline* [title word]’ yields only six entries, only five [2, 3, 4, 5, 6] of which are real treatment guidelines and only an increase of three from 2013. At least some of the cachexia guidelines provide advice for the everyday care of cancer cachectic patients [2, 5]. This disparity in publication activity contrasts with the relative impact of cachexia vs. obesity on mortality. For example, in a cohort of 843 020 patients who had a diagnosis of cardiogenic shock, after multivariate adjustment, cachexia was associated with the highest mortality (40.4%) compared to obesity (26.6%) or being overweight (20.7%) [7].

Similar to Haehling and Anker's [1] conclusions, over a decade later, there is still a large mismatch in the amounts of public research support for these two medical conditions, which is likely a contributing factor to the ongoing publication imbalance. The allocation of research funding provides a clear indication of the field's current standing. Using NIH RePORTER (https://reporter.nih.gov), an advanced text search query, of ‘cachexia’ in Title/Abstract/Terms and fiscal‐year as active projects (search date 12.02.26) the total cost (including subproject costs) of allocated funding totals $58.36 million. When replicating this search with the Title/Abstract/Terms as ‘obesity’, the allocated funding totals $3.14 billion. The ratio of funding allocation is thus 54:1 in favour of obesity studies. Evidently, obesity is much more common than cachexia, and the number of obese individuals is increasing rapidly worldwide; thus, its overall long‐term health impact is much greater. However, research into cachexia and its impact on mortality remains an unmet need. Arguably, cachexia exerts a larger effect on mortality for shorter‐term outcomes than obesity [1]. This confirms that both perception and recognition biases are still prevalent in the research community, the public and hence also among healthcare providers and politicians as to what is important in healthcare [1]. The substantial clinical overlap between advanced ageing/frailty and cachexia, particularly in the domains of involuntary weight loss, sarcopenia, anorexia and systemic inflammation, may contribute to this bias. Therefore, both raising awareness activities and focused research are needed to delineate age‐associated physiological decline from a discrete, syndrome‐mediated catabolic states, ensuring that distinctions with differing prognostic and therapeutic implications are clearly conceptualised, operationalised and communicated across stakeholder groups.

The *Journal of Cachexia, Sarcopenia and Muscle* (*JCSM*) publishes both research and clinical material related to cachexia and sarcopenia, as well as studies of body composition and its physiological and pathophysiological changes during the lifespan and in response to different illnesses from all fields of the life sciences. As a specialist journal, *JCSM* has helped to fill this important gap in publications. The journal was first published in September 2010 and subsequently indexed in PubMed and PubMed Central. *JCSM* has achieved an impressive impact factor of 9.1 and CiteScore of 15.6 as of January 2026. The journal has published 251 manuscripts. Of these publications, it is interesting to see that the three most cited articles in the past 2 years stem from different areas: muscle composition in obesity [8], sarcopenia and mild cognitive impairment [9] and sarcopenia states in older adults [10]—all three papers cited over 40 times each. Obviously, not all researchers working in the field of tissue wasting use the term cachexia. Interestingly, when exploring the addition of the title words ‘sarcopenia’ (*n* = 15 807) and ‘wasting’ (*n* = 4450) to the PubMed search, this improves the publication ratio in 2025 to 1:6 (cachexia or sarcopenia or wasting) vs. obesity. Although this ratio has improved from 2013 (1:10), there remains a substantial unmet need for research into wasting disorders (cachexia, sarcopenia, wasting). Notably however, research in sarcopenia specifically has increased substantially from 2013 to 2025, in a further PubMed title/abstract search of ‘sarcopenia [title/abstract] AND obesity [title/abstract]’ numbers have increased significantly (*n* = 3002) since 2013 with the largest annual number in 2025 (*n* = 563). This may suggest that growing scholarly interest in the field is associated with the recent rise in GLP‐1 receptor agonist prescriptions for obesity, as well as emerging research examining their potential relationship with reductions in lean muscle mass. This represents an important area for further investigation.

When considering that sarcopenia and cachexia exist on a continuum of muscle wasting disorders, where sarcopenia is known to act as a precursor or component of cachexia this begs the question—should there be a greater research focus on cachexia given its impact on mortality? We would argue that the answer is unequivocally yes. The relative scholarly inattention to cachexia stands in stark contrast to the sustained scientific, clinical and commercial mobilisation around obesity. In the current healthcare and research landscape, substantial scientific, policy and commercial attention has been directed towards conditions such as obesity, supported by highly profitable prevention and treatment markets. By contrast, disorders that are less amenable to curative or technology‐driven interventions may be less visible and attract comparatively little investment. Considering cachexia often affects patients with limited life expectancy, this may contribute to its relatively lower prioritisation within research funding, clinical innovation and public health discourse. Yet, its impact on quality of life, morbidity, mortality and healthcare utilisation is profound.

Redressing this imbalance requires deliberate action to leverage more public funding for cachexia research including a strategic approach to capacity building for cachexia research from PhDs onwards. Furthermore, through investment in the ecosystem required to design and deliver clinical translational research in cachexia recruitment pathways, fora for knowledge exchange and profile‐raising activities could be enhanced. Addressing cachexia with more seriousness, greater urgency and improved research infrastructure is scientifically warranted and a matter of equity for some of the most vulnerable patients in our care.

## Funding

This work was supported by Northern Ireland Kidney Research Fund (R229NUR).

## Data Availability

Data sharing not applicable to this article as no datasets were generated or analysed during the current study.
